# Recent progress in the synthesis of metal–organic frameworks

**DOI:** 10.1088/1468-6996/16/5/054202

**Published:** 2015-09-25

**Authors:** Yujia Sun, Hong-Cai Zhou

**Affiliations:** Department of Chemistry, Texas A&M University, College Station, TX 77842-3012, USA

**Keywords:** metal–organic frameworks, solvothermal synthesis, microwave-assisted synthesis, electrochemical synthesis, post-synthetic modification, post-synthetic exchange

## Abstract

Metal–organic frameworks (MOFs) have attracted considerable attention for various applications due to their tunable structure, porosity and functionality. In general, MOFs have been synthesized from isolated metal ions and organic linkers under hydrothermal or solvothermal conditions via one-spot reactions. The emerging precursor approach and kinetically tuned dimensional augmentation strategy add more diversity to this field. In addition, to speed up the crystallization process and create uniform crystals with reduced size, many alternative synthesis routes have been explored. Recent advances in microwave-assisted synthesis and electrochemical synthesis are presented in this review. In recent years, post-synthetic approaches have been shown to be powerful tools to synthesize MOFs with modified functionality, which cannot be attained via *de novo* synthesis. In this review, some current accomplishments of post-synthetic modification (PSM) based on covalent transformations and coordinative interactions as well as post-synthetic exchange (PSE) in robust MOFs are provided.

## 1. Introduction

Metal–organic frameworks (MOFs), also known as porous coordination polymers (PCPs) or porous coordination networks (PCNs), are a rather new class of porous crystalline materials consisting of metal ions or clusters and organic linkers [1–3]. Due to their tunable structure, porosity and functionality, MOFs have attracted considerable attention during the past two decades for applications in many areas, including gas storage [4–8], gas separation [9, 10], catalysis [11–18], sensing [19–22], light harvesting [23], and optical luminescence [24, 25].

In the last few years, considerable efforts have been made to synthesize MOFs. By judicious choice of inorganic joints and organic struts, MOFs with various structures and functionalities have been successfully synthesized [26–28]. So far, MOFs have been generally synthesized from isolated metal ions and organic linkers under hydrothermal or solvothermal conditions via conventional electrical heating on small scales. Recently, the development of the precursor approach and kinetically tuned dimensional augmentation strategy [29] provides more possibilities to obtain novel MOFs with new structures and interesting properties. In order to accelerate the crystallization process and generate uniform crystals with reduced size, many alternative synthesis routes have been investigated, such as microwave-assisted synthesis [30–33], electrochemical synthesis [34–36], sonochemical synthesis [37, 38], mechanochemical synthesis [39, 40] and spray-drying synthesis [41–44]. These methods provide possibilities to synthesize MOFs in a shortened time and with higher quality, which is favorable for industrial applications of MOFs.

Functionality plays a significant role in MOF chemistry since the access to a wide range of potential applications of MOFs depends heavily on the possibility to integrate various chemical functionality into MOFs. However, introducing functionality into MOFs via *de novo* synthesis is not feasible in some cases due to a series of challenges such as limited linker solubility, thermal stability, chemical stability, functional group compatibility, and undesired interference between metal ions and linker functional moieties during MOF assembly. To address these issues, post-synthetic approaches [46–50] have been investigated to functionalize preformed MOFs, including post-synthetic modification (PSM), post-synthetic deprotection (PSD), and post-synthetic exchange (PSE). The achievements in post-synthetic approaches add an additional dimension to the synthetic variability and increase the scope of chemical functionality of MOFs.

This review highlights recent development in the synthesis of MOFs for a wide variety of applications. To begin, we will discuss several synthesis routes towards MOFs, including conventional solvothermal synthesis, microwave-assisted synthesis, and electrochemical synthesis. The precursor approach and kinetically tuned dimensional augmentation strategy will be presented in this part. However, other synthesis methods such as mechanochemical synthesis, sonochemical synthesis, and spray-drying synthesis are beyond the scope of this review. Then we will talk about post-synthetic approaches as useful tools to modify the functionality of preassembled MOFs. In this section, some current work on PSM based on covalent transformations and coordinative interactions as well as PSE implemented in robust MOFs will be presented. Finally, a short summary and comments on future directions will be provided.

## 2. Synthesis routes

### 2.1. Conventional solvothermal synthesis

**Figure 1.**
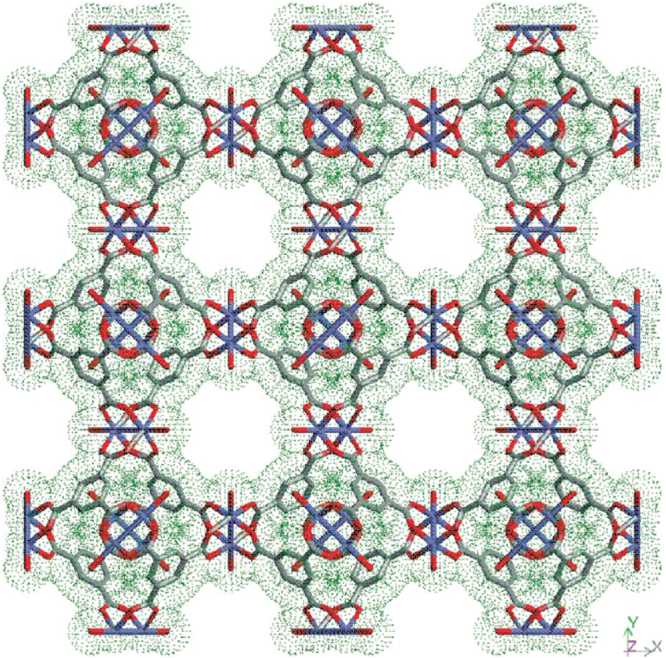
The structure of HKUST-1 viewed down the [100] direction, showing nanochannels with fourfold symmetry. Copper atoms, carbon atoms and oxygen atoms are shown in blue, grey and red. Reprinted with permission from Chui *et al* [45]. Copyright 1999 American Association for the Advancement of Science.

**Figure 2.**
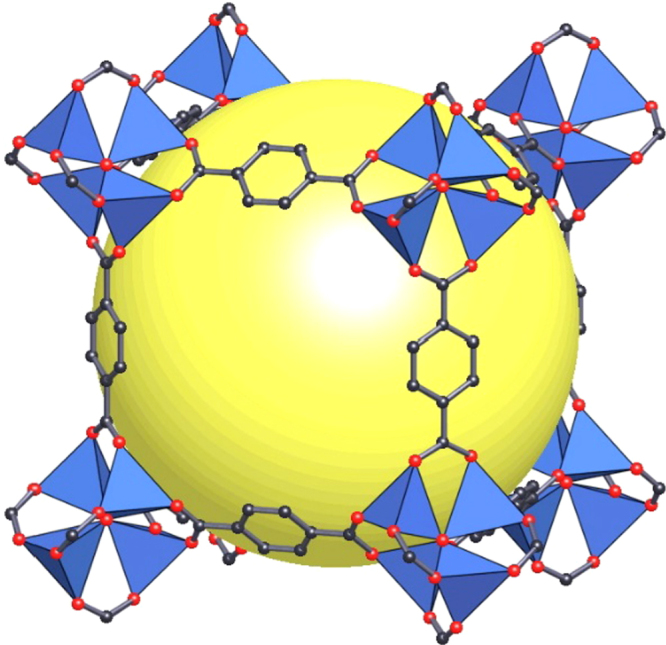
The structure of MOF-5 shown as Zn_4_O tetrahedra (blue polyhedra) joined by benzene dicarboxylate linkers (O: red and C: black) to give an extended 3D cubic framework. Reprinted with permission from Kaye *et al* [51]. Copyright 2007 American Chemical Society.

In general, MOFs have been synthesized under solvothermal conditions via conventional electrical heating. The self-assembling process of MOFs usually starts from isolated metal ions and organic linkers. In 1999, two representative MOFs, HKUST-1 [45] and MOF-5 [52] were reported, symbolizing a benchmark in MOF chemistry. In HKUST-1, Cu paddlewheel secondary building units (SBUs) are coordinated via 1,3,5-benzenetricarboxylate (BTC) to form three-dimensional porous cubic networks (figure 1). On the other hand, MOF-5, with the chemical formula of Zn_4_O(BDC)_3_·(DMF)_8_(C_6_H_5_Cl) where BDC stands for terephthalic acid and DMF for dimethylformamide, consists of Zn_4_O clusters connected to ditopic linear BDC linkers (figure 2). With a similar synthetic method, many representative MOFs exhibiting interesting features have been obtained, such as MIL-53 [53–57], MIL-100 [58–65], MIL-101 [58, 59, 61, 66, –70], MOF-74 [71–74], UiO-66 [75–77] and PCN series [78–85].

**Figure 3.**
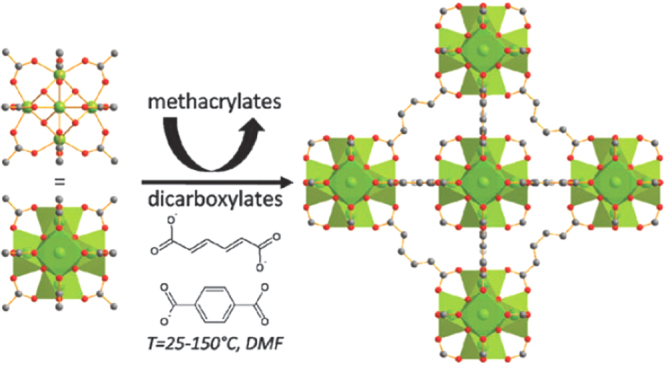
Schematic view of the synthesis of zirconium dicarboxylate MOF starting from Zr_6_ methacrylate oxoclusters. Metal polyhedral, carbon atoms and oxygen atoms are shown in green, grey and red, respectively. Reprinted with permission from Guillerm *et al* [86]. Copyright 2010 Royal Society of Chemistry.

An alternative route to synthesize MOFs is the use of prebuilt inorganic building blocks. The structures and functions of these preformed polynuclear coordination complexes are similar to or the same with the inorganic bricks of the MOF. For example, MIL-88 and MIL-89 were synthesized by replacement of the monocarboxylate (acetate) ligand of a trinuclear oxo-bridged iron(III) acetate by dicarboxylate moieties (fumarate, 2,6-naphthalate, and trans,trans-muconate) [87, 88]. Also, the same group reported the synthesis of porous zirconium dicarboxylates with the UiO-66 architecture by using zirconium methacrylate oxocluster [Zr_6_O_4_(OH)_4_(OMc)_12_] (OMc = CH_2_ = CH(CH_3_)COO) as the precursor (figure 3) [86]. Similarly, this precursor approach has been applied in the synthesis of MOF-5. Starting from [M_4_(*μ*_4_-O)(OAc)_6_], MOF-5(Zn) and MOF-5(Be) were obtained. In addition, cobalt oxopivalate containing two [Co_4_O]^6+^ building units was used to generate MOF-5(Co) [89]. The introduction of metals other than zinc added new features to IRMOFs, such as magnetic properties in MOF-5(Co).

**Figure 4.**
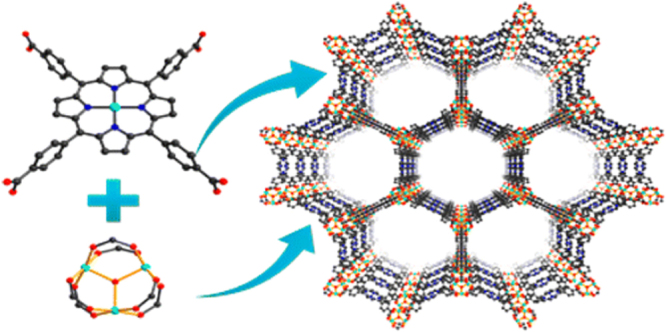
The structure of PCN-600(M) synthesized from metalloporphyrin (C: black, N: blue, O: red, metal: cyan) and preformed metal cluster (C: black, O: red, metal: cyan). Reprinted with permission from Wang *et al* [90]. Copyright 2014 American Chemical Society.

**Figure 5.**
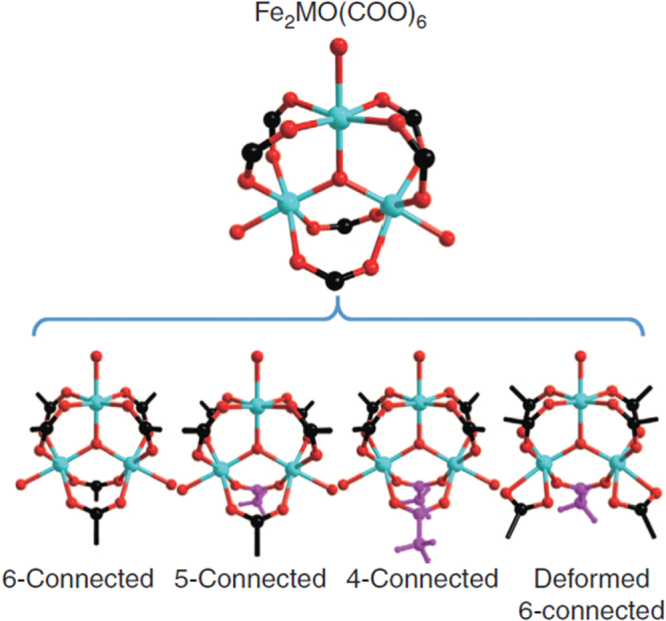
Four different connecting modes of the [Fe_2_M(*μ*_3_-O)] cluster (Fe and M: cyan, O: red). Carboxylates on ligands and terminal acetates are represented by black and purple, respectively. Reprinted with permission from Feng *et al* [29]. Copyright 2014 Nature Publishing Group.

Recently, our group reported the synthesis of a series of mesoporous metalloporphyrin-based MOFs, namely PCN-600(M) (M = Mn, Fe, Co, Ni, Cu) by using preassembled [Fe_3_O(OOCCH_3_)_6_] building block (figure 4) [90]. PCN-600 exhibited a one-dimensional channel as large as 3.1 nm and experimental pore volume of 1.80 cm^3^ g^−1^ as well as very high chemical stability. PCN-600(Fe) has been proved as an active peroxidase mimic to catalyze the co-oxidation reaction. Our group also presented a kinetically tuned dimensional augmentation synthetic route to prepare highly crystalline and robust Fe-MOF with preformed inorganic building blocks [Fe_2_M(*μ*_3_-O)(CH_3_COO)_6_] (M = Fe^2+,3+^, Co^2+^, Ni^2+^, Mn^2+^, Zn^2+^) [29]. By rationalizing the process of MOF growth from both a thermodynamic and a kinetic perspective, large single crystals of 34 different Fe-MOFs with different ligands and various connecting modes of the cluster were obtained (figure 5). Among them, PCN-250(Fe_2_Co) exhibited high volumetric uptake of methane and hydrogen as well as stability in water and aqueous solutions with a wide range of pH values.

### 2.2. Microwave-assisted synthesis

Microwave irradiation has been used to provide energy for the growth of MOFs. Microwave-assisted synthesis is based on the interaction between electromagnetic waves and mobile electric charges, such as polar solvent molecules or ions in the solution. The advantages of this method include high efficiency, phase selectivity, particle size reduction, and morphology control. Due to the overwhelming amount of related literature [32, 92–96], we will mainly focus on some recent examples here.

Cr-MIL-101 is one of the most widely studied MOFs because of ultrahigh surface area and pore volume as well as high thermal and chemical stability, which makes it attractive for various applications, such as gas separation, energy storage, drug delivery and heterogeneous catalysis [58, 59, 66, 69, 70]. In 2011, nano-sized Cr-MIL-101 crystals were obtained by microwave heating at 210 °C [97]. The study of the effect of water concentration and pH on MOF growth revealed that the size of crystals decreases with the increase of water concentration and pH value. In an optimized condition, Cr-MIL-101 with the size of 50 nm was generated easily and efficiently. Similarly, Cr-MIL-101 with size of about 100 nm was synthesized by Zhao *et al* [98], which adsorbed benzene up to 16.5 mmol g^−1^ at 288 K and 56.0 mbar.

**Figure 6.**
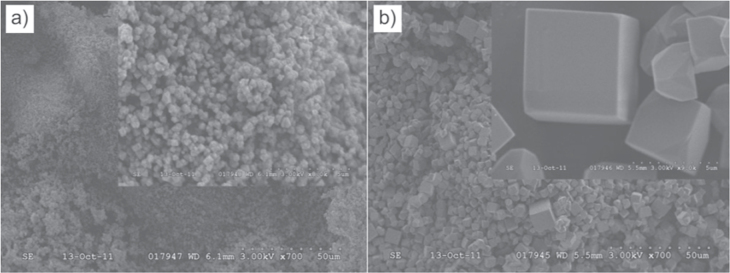
Scanning electron microscopy (SEM) images of (a) CPM-5M and (b) CPM-5(OV) at two different magnifications. Reprinted with permission from Sabouni *et al* [91]. Copyright 2012 WILEY-VCH Verlag GmbH & Co. KGaA, Weinheim.

In 2012, microwave-assisted synthesis of CPM-5 was first reported by Sabouni *et al* [91] (figure 6). This rapid and facile method enabled the generation of CPM-5 with a high surface area of 2187 m^2^ g^−1^ in about 10 min, showing high carbon dioxide uptake. Compared to the microwave-assisted approach, the conventional solvothermal method requires several days for crystallization and the surface area of the product is only 580 m^2^ g^−1^. Following this work, the same group investigated the adsorption equilibrium and diffusion of carbon dioxide in CPM-5 by a volumetric approach at 273 K, 298 K and 318 K and gas pressures up to 105 kPa [99]. Interestingly, this crystalline porous material showed selective adsorption of carbon dioxide over nitrogen, which can be applied for separation of carbon dioxide from flue gas.

**Figure 7.**
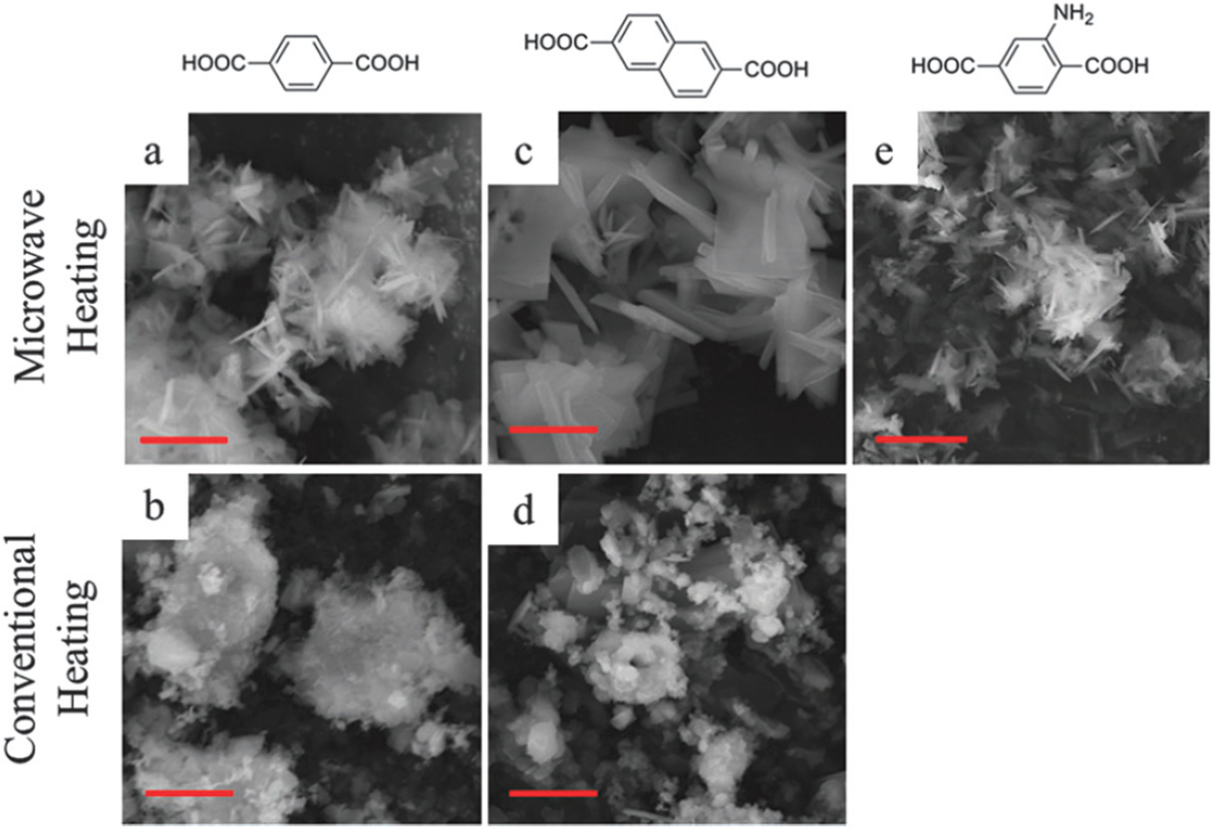
SEM images of (a) MIL-140A-MW, (b) MIL-140A-CE, (c) MIL-140B-MW, (d) MIL-140B-CE and (e) MIL-140A-NH_2_-MW (scale bar = 3 mm). Reprinted with permission from Liang *et al* [100]. Copyright 2013 Royal Society of Chemistry.

In recent years, Zr-based MOFs have attracted great attention due to exceptionally high thermal, hydrothermal and chemical stability [101]. The ultrahigh stability of Zr-based MOFs mainly comes from the strong coordinative interactions between Zr(IV) ions of high charge density and the oxygen atoms of organic linkers. Liang *et al* [100] reported the synthesis of MIL-140 by the microwave-assisted solvothermal method in 2013. They obtained products with purer phase and higher quality in significantly (>95%) less time than the conventional electrical heating method (figure 7). UiO-66, a prototype of Zr-based MOFs, was synthesized by Ren *et al* [102] in 2014. Using the microwave-assisted method, highly crystalline UiO-66 octahedral shaped crystals were obtained in a short reaction time of 5 min and showed hydrogen storage capacity of 1.26 wt.%.

### 2.3. Electrochemical synthesis

The first example of electrochemical synthesis of MOFs is HKUST-1, which was reported in 2005 by researchers at BASF [103], aiming to exclude anions for large-scale production of MOFs. Since then, this synthesis route has been widely applied in MOF chemistry, including the synthesis of Zn-based MOFs, Cu-based MOFs, and Al-based MOFs [104]. In this part, we would like to discuss some recent progress in electrochemical synthesis of MOF thin films, which can be useful in sensing and electrochemical devices.

**Figure 8.**
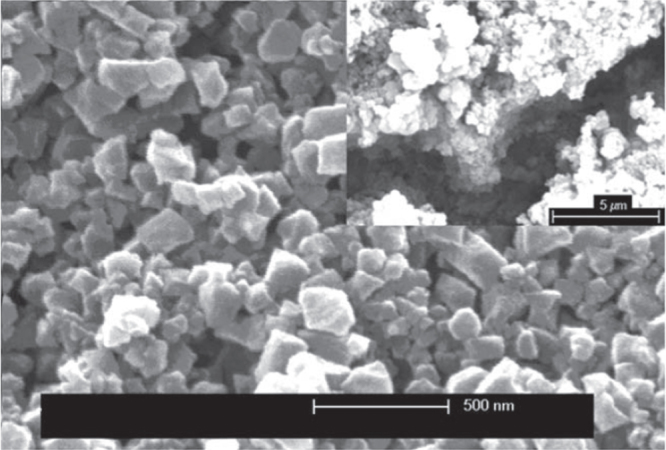
Layer of Fe-BTC MIL-100(Fe) synthesized at 190 °C with solution A on the Fe substrate. Reprinted with permission from Campagnol *et al* [105]. Copyright 2013 Royal Society of Chemistry.

In 2013, synthesis of MIL-100(Fe) by electrochemical deposition under high temperature and high pressure was reported for the first time by Campagnol *et al* [105]. Solution A containing 1,3,5-benzenetricarboxylic acid (H_3_BTC) in a 2:1 ethanol:Milli-Q water solvent mixture was heated in a high temperature, high pressure (HT-HP) electrochemical (EC) cell. Using Fe as the anode, MIL-100(Fe) was generated at various temperatures (110–190 °C) and current densities (2–20 mA cm^−2^) both as crystals in the solution and as a coating on the top of pure iron substrates (figure 8). Taking HKUST-1 as an example, they also showed that this HT-HP cell can be used to modify the crystal morphology of MOFs.

**Figure 9.**
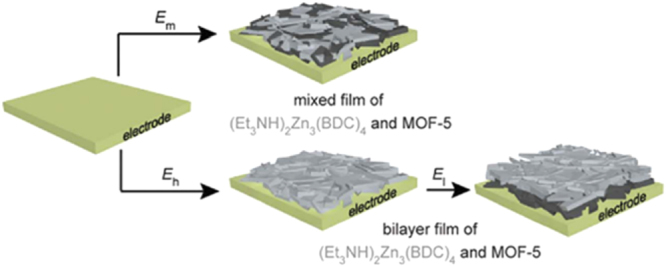
Schematic view of the formation of a biphasic mixed film at (cathodic) potential, *E*_l_ (*E*_l_ < *E*_m_ < *E*_h_). Reprinted with permission from Li *et a*l [106]. Copyright 2014 Royal Society of Chemistry.

Potential-modulated formation of biphasic thin films of MOFs was reported in 2014 by Li *et al* [106]. The authors originally proposed using triethylammonium as a probase to form trimethylamine and facilitate the cathodic electrodeposition of MOFs. With the presence of high concentration of triethylammonium, no Zn deposition was observed and the anionic framework (Et_3_NH)_2_Zn_3_(BDC)_4_ was formed. This is mainly caused by etching of the Zn layer by triethylammonium and the low effective concentration of trimethylamine to induce the generation of MOF-5 since trimethylamine buffers the pH. By reducing the concentration of triethylammonium, formation of (Et_3_NH)_2_Zn_3_(BDC)_4_ at higher potential and MOF-5 at lower potential was accomplished. In addition, synthesis of mixed film and bilayer film was achieved by controlling the potential (figure 9). This report clearly demonstrated the potential of using electrochemical methods to synthesize heterogeneous multiphasic and multilayered MOF thin films and membranes.

Although electrochemical synthesis has been demonstrated as an active route for generation of MOF layers, a limitation of anodic electrodeposition, which consists of anodic generation of the metal ions required for MOF formation in a solution containing organic linkers, is that only MOFs with the same metal as the substrate can be synthesized. Campagnol *et al* [107] recently presented the preparation of MOF films with metal oxide as the substrate by anodic electrodeposition. With this method, Tb-BTC on Al and Zn-BTC doped with Tb(III) on zinc were successfully synthesized without using expensive rare earth substrates. The luminescent Tb-containing MOF films showed efficiency in detecting 2,4-dinitrotoluene (DNT), a by-product of 2,4,6-trinitrotoluene (TNT).

**Figure 10.**
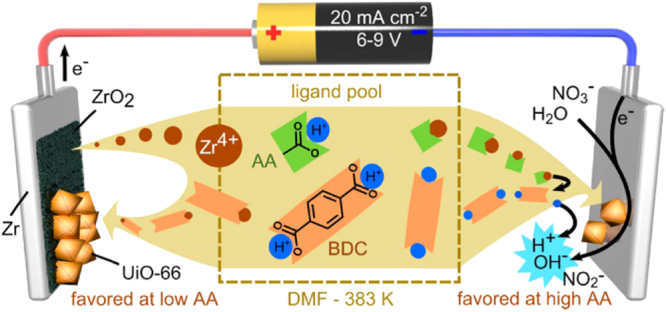
Scheme of the anodic and cathodic electrochemical deposition mechanisms. Reprinted with permission from Stassen *et al* [108]. Copyright 2015 American Chemical Society.

Recently, Stassen *et al* [108] reported both the anodic and cathodic electrochemical film deposition of UiO-66 with zirconium foil as the only metal source (figure 10). First, the synthesis solution containing BDC:HNO_3_:H_2_O:AA:DMF = 1:2:4:5/10/50:130 was prepared and heated to 383 K. Then, film deposition was accomplished by applying a current of 80 mA at 383 K. Superior adhesion of the MOF layer onto the zirconium substrate was observed for anodic deposition due to the formation of an oxide bridging layer. On the other hand, cathodic deposition possessed the advantage of wide substrate flexibility. This synthesis method showed patterned deposition capability and allowed the straightforward utilization of UiO-66 in a miniaturized sorbent trap for applications such as online analytical sampling as well as concentration of dilute volatile organic complexes.

## 3. Functionality modification

Functionality plays an important role in MOF chemistry since the access to a wide range of potential applications of MOFs depends on the possibility of incorporating various chemical functionality into MOFs to a great extent. However, in some cases, introducing functionality into MOFs via direct synthesis approaches is not suitable due to several challenges such as limited linker solubility, thermal stability, chemical stability, functional group compatibility, and undesired interference between metal ions and linker functional moieties during MOF assembly. To address these issues, post-synthetic approaches were investigated to modify the functionality of preassembled MOFs, such as post-synthetic modification (PSM) [46–48], post-synthetic deprotection (PSD) [109], and post-synthetic exchange (PSE) [49, 50]. The achievements in post-synthetic approaches add an additional dimension to the synthetic variability and increase the scope of chemical functionality that can be integrated into MOFs. Through these methods, functionality of specific interest can be introduced into MOFs while maintaining the structural integrity, which is difficult to achieve via direct synthesis of MOFs. In this section, we will primarily focus on current accomplishments in PSM and PSE.

### 3.1. PSM

**Figure 11.**
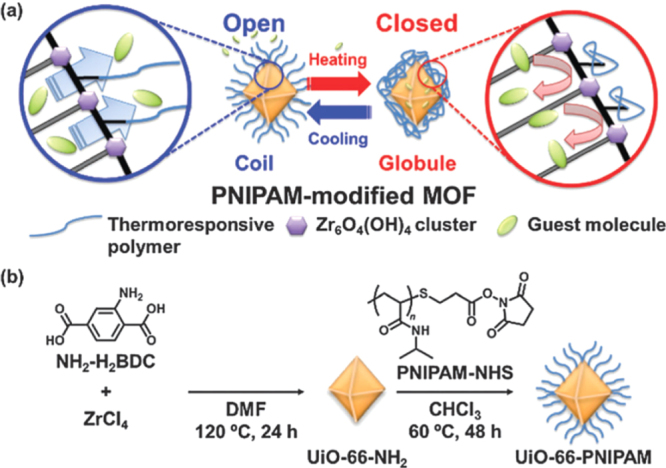
(a) Schematic view of controlled release by PNIPAM-modified MOF. (b) Preparation of PNIPAM-modified MOF. Reprinted with permission from Nagata *et al* [124]. Copyright 2015 Royal Society of Chemistry.

One of the most extensively applied PSM approaches is the modification of organic linkers via chemical reactions with the preservation of lattice structure. So far, various kinds of covalent transformations have been successfully investigated by a number of researchers to modify preassembled MOFs, such as amide coupling [110–112], imine condensation [113–115], urea formation [111, 116], salicylaldehyde condensation [117], N-alkylation [11], click reactions [118–121], bromination [112], and protonation [122, 123]. Many of these transformation reactions have been implemented in amino-functionalized MOFs. For example, Nagata *et al* [124] reported a surface- selective PSM method to modify UiO-66-NH_2_ with a thermoresponsive polymer poly(N-isopropylacrylamide) (PNIPAM). The conformational change of PNIPAM with the temperature led to an ‘open’ state at lower temperature and a ‘closed’ state at higher temperature (figure 11). This smart UiO-66-PNIPAM showed promising applications in controlled release of the guest molecules such as resorufin, caffeine, and procainamide.

**Figure 12.**
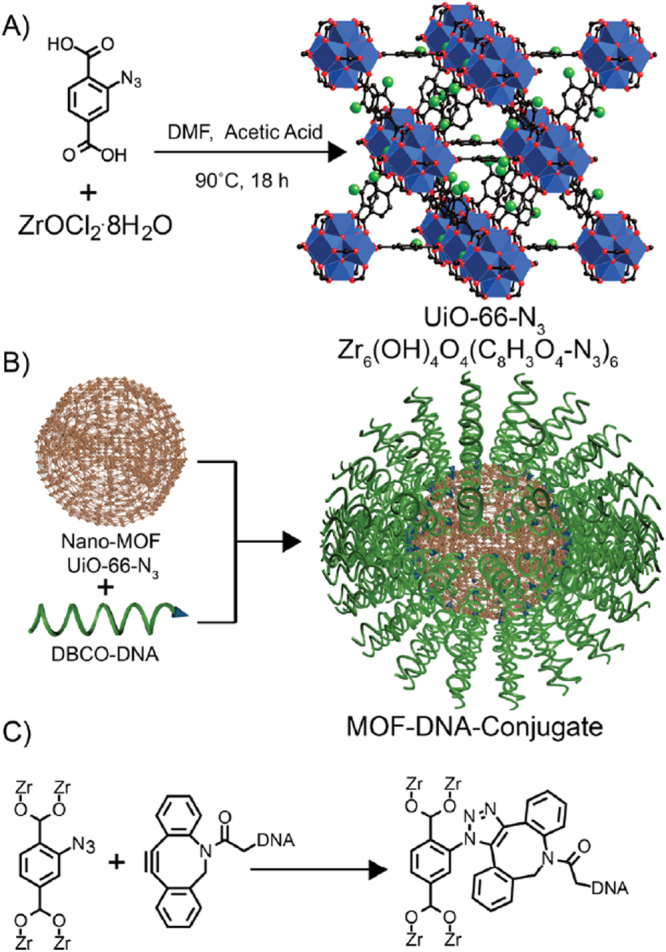
(a) Synthesis of UiO-66-N_3_ nanoparticles. (b) DNA functionalization of UiO-66-N_3_ nanoparticles. (c) Strain promoted click reaction between the MOF and DNA. Reprinted with permission from Morris *et al* [120]. Copyright 2014 American Chemical Society.

Azide-functionalized MOFs have been utilized to undergo click reactions. In 2014, the first MOF nanoparticle-nucleic acid conjugates were prepared by Morris *et al* [120]. These conjugates were generated by a strain promoted click reaction between DNA modified with dibenzylcyclooctyne and azide-functionalized UiO-66-N_3_ to covalently functionalize the surface of the MOF with oligonucleotides while preserving the structure of the framework (figure 12). They exhibited higher stability and cellular uptake in aqueous NaCl than unfunctionalized MOF particles of similar size. This work presented the synthesis of a new class of nanostructures for potential applications in chemistry, materials science and biology.

**Figure 13.**
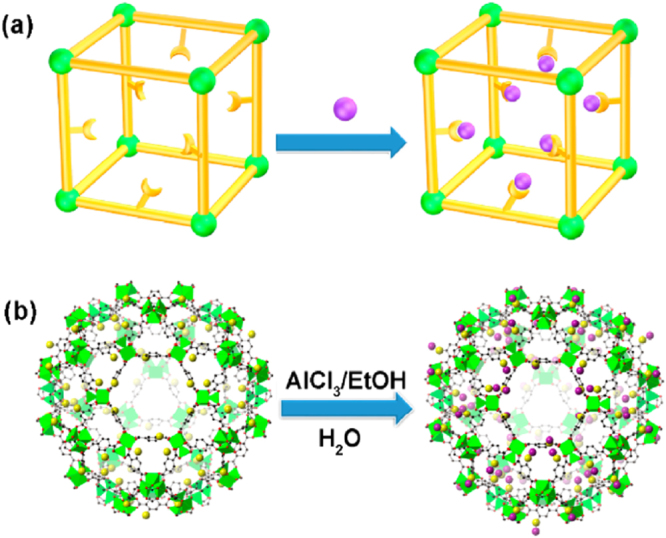
(a) Schematic view of the synthesis of Lewis acid@Br⊘nsted acid MOF. (b) Schematic view of the synthesis process for MIL-101-Cr-SO_3_H·Al(III). Reprinted with permission from Li *et al* [125]. Copyright 2015 American Chemical Society.

In addition to covalent bond formation, some PSM approaches are based on coordinative interactions. For example, Li *et al* [125] reported the synthesis of a Lewis acid@Br⊘nsted acid MOF, named MIL-101-Cr-SO_3_H·Al(III). This MOF was prepared by the reaction of AlCl_3_ with MIL-101-Cr-SO_3_H in ethanol followed by water treatment. Because of strong Lewis acidity, Al(III) centers were successfully incorporated into the Br⊘nsted acidic MIL-101-Cr-SO_3_H to obtain MIL-101-Cr-SO_3_H·Al(III) (figure 13). The synergy between Lewis acidic Al(III) centers and the Br⊘nsted acidic framework improved the catalytic activity of MIL-101-Cr-SO_3_H·Al(III) in benzylation of mesitylene with benzyl alcohol. Notably, it was revealed that the catalytic performance of this post-synthetically modified MOF exceeded two benchmark zeolite catalysts (H-Beta and HMOR). This work provided a new way to enhance the activity of MOFs as heterogeneous catalysts.

### 3.2. PSE

Beyond above widely studied PSM approaches, many conceptually distinct post-synthetic routes have emerged in recent years. Post-synthetic exchange, also known as building block replacement (BBR) [50, 126], involves the replacement of key structural components of the preassembled MOF. With the development of MOF chemistry, synthesizing MOFs with both excellent stability and desired functionality have been a sought after goal due to the importance in practical applications. Here, we would like to take UiO-66 as an example to present current advances in functionalization of robust MOFs.

**Figure 14.**
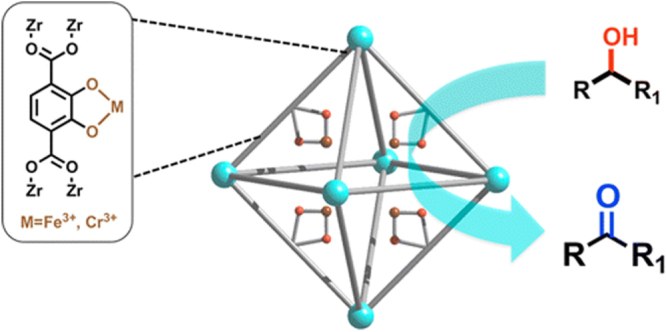
Schematic view of catechol-functionalized UiO-66 after metalation for catalytic oxidation of alcohols to ketones. Reprinted with permission from Fei *et al* [109]. Copyright 2014 American Chemical Society.

**Figure 15.**
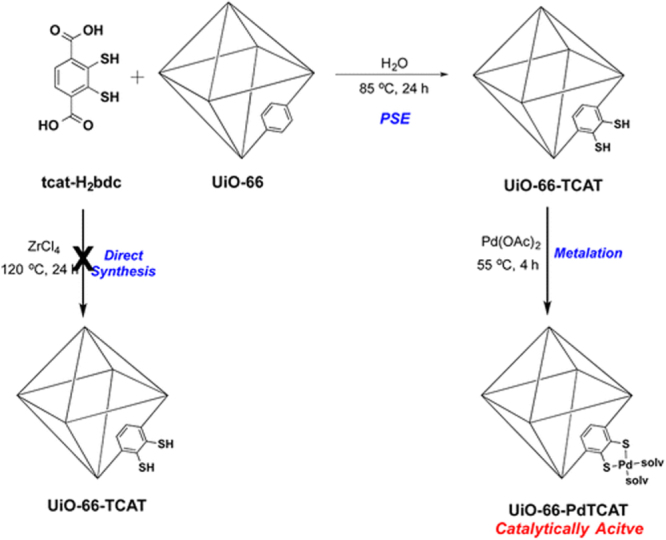
Schematic view of the synthesis of UiO-66-TCAT and UiO-66-PdTCAT. Reprinted with permission from Fei *et al* [128]. Copyright 2015 American Chemical Society.

Solvent-assisted linker exchange (SALE) is one of the most widely used PSE approaches to tune the functionality of UiO-66. For example, BDC linkers in UiO-66 were exchanged by various flexible alkanedioic acids (AD) by Hong *et al* [127] to generate a series of modified UiO-66-ADn derivatives (ADn: HOOC-(CH_2_)*_n_*-COOH, *n* = 4, 6, 8, and 10). During the exchange process, one BDC linker was substituted by two AD to form UiO-66 modified with pendant carboxylic groups. This functionalized UiO-66 showed enhanced selectivity for CO_2_ uptake over CH_4_, which is promising for separation of CO_2_ from landfill gas. In 2014, Fei *et al* [109] reported the modification of UiO-66 with catechol functionality by SALE in a DMF/H_2_O solution of CATBDC for 2 days at 85 °C. After metalation, Cr-metalated MOFs were synthesized and showed high activity in catalytic oxidation of alcohols to ketones (figure 14). The same group also obtained thiocatechol-functionalized UiO-66 [128]. After metalation reaction with Pd(OAc)_2_ at 55 °C for 4 days, UiO-66-PdTCAT was synthesized (figure 15), which is efficient in regioselective functionalization of sp^2^ C−H bond. Recently, Nickerl *et al* [129] successfully incorporated dihydro-1,2,4,5-tetrazine-3,6-dicarboxylate into UiO-66 by linker exchange. The obtained tetrazine functionalized UiO-66 was studied as an optical sensor to detect oxidative agents such as nitrous gases. Interestingly, reversible oxidation and reduction of the tetrazine unit in UiO-66 led to a significant color change of the MOF.

Transmetalation at inorganic nodes represents another kind of PSE route. In 2012, Kim *et al* [130] reported the synthesis of the first Ti(IV) analogue of UiO-66(Zr) by transmetalation. UiO-66(Zr/Ti) was obtained by exposing UiO-66(Zr) to DMF solutions of Ti(IV) salts for 5 days at 85 °C, which was confirmed by positive-ion ATOFMS spectra for the presence of Ti(IV) ion. Followed by this work, Hon *et al* [131] demonstrated that transmetalation of Zr by Ti in UiO-66 could lead to almost doubled CO_2_ uptake due to decreased pore size and increased adsorption enthalpy. More recently, Lee *et al* [132] reported that UiO-66(Zr/Ti) could undergo photocatalytic CO_2_ reduction to form HCOOH upon visible light irradiation in the presence of 1-benzyl-1,4-dihydronicotinamide (BNAH) and triethanolamine (TEOA). Notably, mixed-ligand UiO-66(Zr/Ti) exhibited better catalytic performance due to formation of new energy levels in the band structure of the framework.

**Figure 16.**
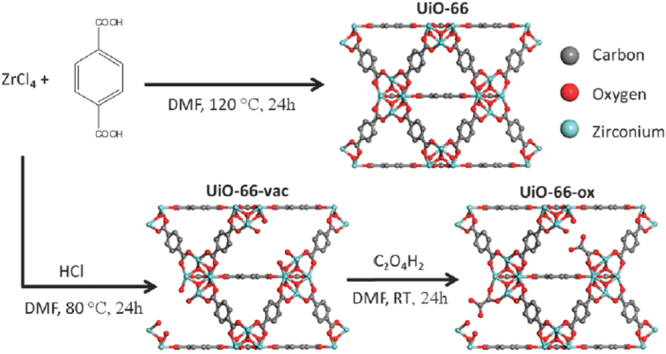
Scheme of the synthesis of UiO-66, UiO-66-vac, and UiO-66-ox. RT stands for room temperature. Reprinted with permission from DeCoste *et al* [135]. Copyright 2015 Royal Society of Chemistry.

Moreover, solvent-assisted linker incorporation (SALI) has been applied in UiO-66, which is facilitated by the missing-linker defects in UiO-66 [133, 134]. Recently, DeCoste *et al* [135] demonstrated the modification of UiO-66 with oxalic acid for broad-spectrum removal of toxic chemicals, including ammonia and cyanogen chloride. During the modification process, the vacant sites of UiO-66 were incorporated by oxalic acid, where one carboxylate group coordinates to the Zr_6_ cluster and the other is free in the pore (figure 16).

## 4. Conclusions

In previous sections we talked about recent advances in the synthesis of MOFs. With the goal of creating new compounds and structures with intriguing properties, this field has been developed and is expanding rapidly. In general, MOFs have been synthesized from isolated metal ions and organic linkers under hydrothermal or solvothermal conditions via conventional electrical heating. The development of precursor approach and kinetically tuned dimensional augmentation strategy diversifies this field and facilitates the discovery of MOFs with new structures and interesting properties. So far, many alternative routes have been established, including microwave-assisted synthesis, electrochemical synthesis, sonochemical synthesis, mechanochemical synthesis and spray-drying synthesis. These approaches have demonstrated to be suitable for some materials, leading to compounds, often under milder reaction conditions and in a short time, with pure phase, reduced particle size, and controlled morphology. However, the reproducibility of these procedures needs to be improved. In addition, only a few unknown compounds have been synthesized by these unconventional routes. In order to obtain novel MOFs, various factors should be considered during the synthesis, such as concentration of starting materials, solvent, pH, reaction temperature, and reaction time. The emerging high-throughput methods provide ideal tools to study MOFs systematically by combining the ideas of parallelization, miniaturization, and automation within the workflow. We expect that the effective combination of high-throughput approaches and various synthesis routes will contribute to optimization of the synthetic procedure and accelerate the discovery of novel MOFs.

On the other hand, post-synthetic approaches have been shown to be useful tools to synthesize MOFs with tuned functionality in the past few years, including PSM, PSD, and PSE. These methods provide possibilities to introduce functionality into MOFs while preserving their structural integrity, which cannot be achieved via *de novo* synthesis due to limited linker solubility, thermal stability, chemical stability, functional group compatibility, and undesired interference between metal ions and linker functional moieties during MOF assembly. The accomplishments in post-synthetic approaches add an additional dimension to the synthetic variability and increase the scope of chemical functionality that can be integrated into MOFs. However, there are some disadvantages of post-synthetic approaches. For example, it is challenging to spatially resolve the distribution of functional groups post-synthetically incorporated into the MOF. As a result, most of the MOF structures are hypothesized. In some cases, the degree to post-synthetically integrate functional groups into MOFs is very limited. In addition, further studies are required to investigate the chemical principles of these specific phenomena, including SALE, SALI, and transmetalation, in order to better guide the synthesis of MOFs with desired functionality. With the development of various synthesis routes and deeper understanding of post-synthetic approaches, we expect that synthesis of robust MOFs with new structures and interesting properties for various practical applications will be achieved in the future.
